# Neurobehavioral Effects of LSVT^®^ LOUD on Auditory-Vocal Integration in Parkinson’s Disease: A Preliminary Study

**DOI:** 10.3389/fnins.2021.624801

**Published:** 2021-02-26

**Authors:** Yongxue Li, Mingdan Tan, Hao Fan, Emily Q. Wang, Ling Chen, Jingting Li, Xi Chen, Hanjun Liu

**Affiliations:** ^1^Department of Rehabilitation Medicine, The First Affiliated Hospital, Sun Yat-sen University, Guangzhou, China; ^2^Department of Rehabilitation Medicine, The First Affiliated Hospital of Zhengzhou University, Zhengzhou, China; ^3^Department of Communication Disorders and Sciences, RUSH University Medical Center, Chicago, IL, United States; ^4^Department of Neurology, The First Affiliated Hospital, Sun Yat-sen University, Guangzhou, China; ^5^Guangdong Province Key Laboratory of Brain Function and Disease, Zhongshan School of Medicine, Sun Yat-sen University, Guangzhou, China

**Keywords:** speech motor control, Parkinson’s disease, LSVT LOUD, auditory feedback, event-related potentials

## Abstract

Individuals with Parkinson’s disease (PD) are impaired in auditory-vocal integration, characterized by abnormal compensatory responses to auditory feedback errors during self-monitoring of vocal production. The present study examined whether auditory feedback control of vocal pitch production in PD can benefit from Lee Silverman voice treatment (LSVT^®^ LOUD), a high effort, intensive speech treatment for hypokinetic dysarthria in PD. Before and immediately after LSVT LOUD, 12 individuals with PD were instructed to produce sustained vowel sounds while hearing their voice unexpectedly pitch-shifted by −200 cents. Their vocal responses and event-related potentials (ERPs) to pitch perturbations were measured to assess the treatment outcomes. A group of 12 healthy controls were one-to-one pair matched by age, sex, and language. Individuals with PD exhibited abnormally enhanced vocal and ERP P2 responses to pitch perturbations relative to healthy controls. Successful treatment with LSVT LOUD, however, led to significantly smaller and faster vocal compensations that were accompanied by significantly larger P2 responses. Moreover, improved vocal loudness during passage reading was significantly correlated with reduced vocal compensations for pitch perturbations. These preliminary findings provide the first neurobehavioral evidence for beneficial effects of LSVT LOUD on impaired auditory-vocal integration associated with PD, which may be related to improved laryngeal motor functions and a top-down modulation of the speech motor network by LSVT LOUD.

## Introduction

Idiopathic Parkinson’s disease (PD) is a neurodegenerative disorder, affecting not only 1.0–1.5% of individuals aged 60 years and older (Marsden, 1994) but also younger individuals aged 20–40 years (Kostic, 2009). During the disease progression, 70–90% of individuals with PD develop hypokinetic dysarthria, characterized by reduced voice loudness, reduced intonation variability, speech dysfluency, and imprecise articulation (Duffy, 2005). These speech disorders are represented by acoustic and perceptual measures of reduced vocal sound pressure level (SPL), shorter sustained phonation, decreased speech intelligibility, and monotonous prosody (Ackermann and Ziegler, 1991; Ho et al., 1998; Sapir et al., 2007; De Keyser et al., 2016), leading to reduced quality of daily life and progressive loss of social communication (Miller et al., 2006).

To date, the neural mechanisms underlying speech disorders in PD remain unclear. A growing body of literature has suggested abnormalities in auditory-vocal integration, characterized by deficits of perceiving errors in voice auditory feedback and regulating vocal motor behaviors, as an important factor contributing to hypokinetic dysarthria in PD (Sapir et al., 2007; Moreau and Pinto, 2019). For example, individuals with PD often overestimate their speech loudness during both reading and conversation (Fox and Ramig, 1997; Ho et al., 2000). These perceptual deficits lead to their abnormalities in auditory-motor control of speech production. For example, individuals with PD were less able to adjust their voice loudness than healthy controls when auditory feedback was altered or distorted by background noise (Ho et al., 1999, 2000). Recent evidence has shown enhanced vocal compensations for perturbations in fundamental frequency (*f*_*o*_) and intensity in individuals with PD when compared to healthy controls (Liu et al., 2012; Chen et al., 2013; Huang et al., 2016). In contrast, they exhibited significantly smaller compensations for speech *F*_1_ perturbations than healthy controls (Mollaei et al., 2016). This discrepancy may be related to the distinct mechanisms underlying the control of *f*_*o*_ and *F*_1_ during speech production (Bouchard et al., 2013). These abnormalities in feedback control process associated with PD also affect sensorimotor learning during speech production, as reflected by reduced adaptive responses to persistent perturbations in voice *f*_*o*_ and speech *F*_1_ (Mollaei et al., 2013; Abur et al., 2018). Together, these findings demonstrate that individuals with PD are impaired in auditory-vocal integration, leading to their deficits in the online detection and correction of auditory feedback errors during self-produced speech.

Given the complexity of the neural bases of hypokinetic dysarthria in PD, however, it is challenging to develop effective rehabilitation approaches. Medical treatments including dopamine therapy and deep brain stimulation (DBS) are effective in improving limb symptoms, but their impact on hypokinetic dysarthria remains a matter of debate (Pinto et al., 2004; Tripoliti et al., 2011; Rusz et al., 2016; Brabenec et al., 2017). Of the behavioral treatment programs, Lee Silverman Voice Treatment (LSVT^®^ LOUD), a high effort, intensive speech treatment with a focus on recalibrating sensorimotor perception of improved vocal loudness (Ramig et al., 1996), has been well documented for its positive beneficial effects on the treatment of hypophonia (Ramig et al., 2001a,b; Sapir et al., 2007; Fox et al., 2012; Sauvageau et al., 2015; Li et al., 2020; Nakayama et al., 2020). This training-induced improvement in vocal loudness can be maintained as long as 12–24 months (Ramig et al., 1996, 2001a; Nakayama et al., 2020). Moreover, beneficial effects of LSVT LOUD on vocal loudness have been shown to spread to other speech subsystems, leading to improvement in pitch variability, articulatory precision, and speech intelligibility (Fox et al., 2002; Sauvageau et al., 2015; Nakayama et al., 2020). However, whether abnormal auditory-motor control of vocal pitch production associated with PD can likewise benefit from LSVT LOUD is not yet known.

Two lines of converging evidence lead us to hypothesize that LSVT LOUD may have an equal potential for generating beneficial effects on impaired auditory-motor integration for vocal pitch regulation associated with PD. Previous studies showed that, in addition to increased vocal loudness, *f*_*o*_ modulation, and articulatory precision, individuals with PD following LSVT LOUD also exhibited improved vocal fold adduction, glottic closure, vocal fold vibratory movements, and increased activity in thyroarytenoid muscle (Fox et al., 2002; Mahler et al., 2015). These physiologic changes in the laryngeal systems are also important for the online control of voice *f*_*o*_, as reflected by the findings of the coordinated movement of the cricothyroid and thyroarytenoid muscles for compensatory adjustments of vocal pitch regulation (Liu et al., 2011a) and increased vocal compensations for pitch perturbations as a result of anesthetization of the vocal folds (Larson et al., 2008). Therefore, improvement of the laryngeal motor functions in individuals with PD following LSVT LOUD may facilitate their neuromuscular control of vocal pitch production. On the other hand, neuroimaging studies of efficacy of LSVT LOUD for hypophonia in PD have shown a functional brain reorganization as reflected by a treatment-induced right shift in the frontal–temporal–parietal regions (Liotti et al., 2003; Narayana et al., 2010). Particularly, improved vocal SPL was predicted by increased activity in the right middle temporal gyrus (MTG), premotor cortex (PMC), and dorsolateral prefrontal cortex (DLPFC), suggesting that LSVT LOUD can improve self-monitoring of speech production by recalibrating auditory-motor integration in a top-down manner (Narayana et al., 2010). In the context of speech motor control, the MTG and PMC are involved in the detection and correction of voice auditory feedback errors (Chang et al., 2013; Kort et al., 2016; Guo et al., 2017), while the DLPFC can exert top-down inhibitory influences on vocal pitch regulation (Liu et al., 2020). Therefore, LSVT LOUD may augment auditory feedback control of vocal pitch production through the top-down modulations of the speech motor systems.

Therefore, the present study sought to investigate whether, and if so, how LSVT LOUD can improve auditory-motor integration for vocal pitch regulation in individuals with PD. Before and immediately after LSVT LOUD, participants were instructed to vocalize the vowel sound /u/ while hearing their voice unexpectedly pitch-shifted downward by 200 cents. In addition to the vocal responses to pitch perturbations, the event-related potentials (ERPs) of N1 and P2 components phase-locked to the pitch perturbations were also obtained to assess the treatment outcomes. These two components are prominently pronounced in the cortical processing of voice pitch perturbations, reflecting the early detection of auditory feedback errors and later cognitive processing of auditory-motor integration (Korzyukov et al., 2012; Guo et al., 2017). For example, larger N1 and P2 responses were elicited by larger size of pitch perturbations (Behroozmand et al., 2009; Liu et al., 2011b; Scheerer et al., 2013). Training-induced decrease of N1 responses and/or increase of P2 responses to pitch perturbations were also found when healthy participants underwent speech perceptual learning or working memory training (Chen et al., 2015; Li et al., 2015; Guo et al., 2017). Previous findings have shown enhanced vocal and/or P2 responses to pitch perturbations in individuals with PD (Liu et al., 2012; Chen et al., 2013; Huang et al., 2016; Mollaei et al., 2016). When instructed to vocalize the vowels to match the pitch target, however, they exhibited decreased vocal compensations and N1 responses but increased P2 responses as compared to when they vocalized with a memory trace of the pitch target learned before the experiment (Huang et al., 2019). Together with the observed beneficial effects of LSVT LOUD on voice *f*_*o*_ modulation (Fox et al., 2002; Mahler et al., 2015; Sauvageau et al., 2015), we hypothesized that LSVT LOUD would likewise lead to decreased vocal compensations, decreased N1 amplitudes, and increased P2 amplitudes in response to pitch perturbations in individuals with PD, reflecting beneficial effects of LSVT LOUD on their impaired auditory-vocal integration.

## Materials and Methods

### Subjects

A group of 12 adults [11 male and one female; mean and standard deviation (SD): 61.92 ± 9.34 years], with a clinical diagnose of idiopathic PD according to the diagnostic criteria of the United Kingdom PD Society Brain Bank (Hughes et al., 1992), participated in the present study (see Table 1). Four of them were native Mandarin speakers and the others were native Cantonese speakers. They overlapped with the cohort of 16 individuals with PD (14 male and two female; 64.06 ± 8.86 years) reported in our previous study (Li et al., 2020) that showed significant improvement of vocal SPL for sustained phonation, paragraph reading, and monolog following LSVT LOUD. Individuals with PD in the present study met the following criteria: no more than mild dementia [Mini-Mental State Examination (MMSE) >26], no other neurological disease, no history of neurosurgical treatment, laryngeal surgery or pathology, and swallowing disorders. The mean disease duration was 6.25 ± 3.72 years; the mean Modified Unified Parkinson’s disease Rating Scale (MDS-UPDRS) was 38.91 ± 13.87; the mean MDS-UPDRS Part II-1 (speech) was 1.58 ± 0.79; and the mean MDS-UPDRS Part III-1 (speech) was 1.75 ± 0.75. The Modified Hoehn and Yahr scores ranged from 2 to 3 with a mean score of 2.5 ± 0.4. Individuals with PD were kept on their antiparkinsonian medication during intensive voice treatment, but they were tested during their off-medication state (i.e., 12 h off anti-PD medication). A group of 12 neurologically normal adults served as healthy controls. They were one-to-one pair matched with individuals with PD by language, sex, and age (11 male; 61.92 ± 9.34 years; *t* < 0.001, d.f. = 11, *p* = 1.000). None of them reported a history of speech, hearing, and neurological disorders. All participants were right-handed, and passed a hearing screening with a threshold of 40 dB hearing level (HL) or less for 250, 500, 1k, 2k, and 4k Hz binaurally. The research protocols were approved by the Institutional Review Board of The First Affiliated Hospital at Sun Yat-sen University, with written informed consent obtained from all participants.

**TABLE 1:** Demographic and clinical characteristics of individuals with PD.

Patients	Age (year)/sex	PD duration (year)	MDS-UPDRS Total (ON)	MDS-UPDRS II-1 (speech)	MDS-UPDRS III-1 (speech)	M-HY
S1	64/M	10	41	1	1	2.5
S2	62/M	3	18	1	1	2
S3	77/M	14	61	3	3	3
S4	70/M	8	44	3	2	3
S5	52/M	3	45	1	1	2.5
S6	63/M	5	34	1	2	2.5
S7	65/M	4	33	1	1	2.5
S8	48/F	5	20	1	2	2
S9	59/M	2	48	2	2	2.5
S10	56/M	3	23	1	1	2
S11	76/M	8	58	2	3	3
S12	51/M	10	42	2	2	2.5

*PD, Parkinson’s disease; MDS-UPDRS, Modified Unified Parkinson’s Disease Rating Scale; M-HY, Modified Hoehn and Yahr Scale.*

### Intensive Voice Treatment

Individuals with PD received intensive voice treatment according to the LSVT LOUD program (Ramig et al., 1995) with one speech language pathologist (the first author, YL) who was globally certified in LSVT LOUD treatment delivery. This program requires four 1-h in-person treatment sessions per week for four consecutive weeks at the hospital, leading to a total of 16 training sessions. Each session consisted of daily exercises that focused on maximum sustained phonation of /a/, maximum *f*_*o*_ range, and reading of functional phrases. Also, per LSVT LOUD protocol, individuals with PD were required to perform daily homework and carry-over tasks. Their dosages of antiparkinsonian medication remained unchanged throughout the training period.

### Procedure

Prior to the present study, individuals with PD performed the vocal loudness tests that consisted of sustained vowel phonation of /a/, reading a passage, and monolog before and immediately after LSVT LOUD. Also, their voice quality was evaluated using the Japanese GRBAS voice scale (Takahashi and Koike, 1976) and their quality of life was evaluated using the Voice Handicap Index (VHI) scale (Jacobson et al., 1997). The results have been reported in our previous study (Li et al., 2020), showing significant improvement of vocal loudness and quality of their voice and life following LSVT LOUD.

In the present study, all participants completed the vocal production experiment based on the frequency-altered feedback (FAF) paradigm (Burnett et al., 1998). Individuals with PD participated in this experiment before and immediately after LSVT LOUD. All participants were instructed to produce sustained phonations of the vowel /u/ for about 4–5 s, during which their voice feedback was pseudo-randomly pitch-shifted downward four times by 200 cents or 2 semitones (200 ms duration; 100 cents = 1 semitone or 5.95% of the frequency change). The first pitch perturbation occurred with a random delay of 1500–2500 ms relative to the vocal onset, and the succeeding pitch perturbations were presented with an inter-stimulus interval of 700–1000 ms. All participants were required to take a break of 2–3 s between consecutive vocalizations to avoid vocal fatigue. They each produced 25 consecutive vocalizations, leading to a total of 100 trials for −200 cents pitch perturbations.

### Apparatus

The experiment was conducted in a sound-attenuated room. In order to minimize the masking effects of air-born and bone-conducted feedback, the recording system was acoustically calibrated by setting the intensity of voice feedback 10 dB SPL higher than that of participant’s vocal output. During the experiment, the voice signals were transduced through a dynamic microphone (DM2200, Takstar Inc.) and sent to an Eventide Eclipse Harmonizer through a MOTU Ultralite Mk3 Firewire audio interface. A MIDI software program (Max/MSP v.5.0 by Cycling 74) was developed to control the Eventide Eclipse Harmonizer to pitch-shift the voice signals. Also, transistor-transistor logic (TTL) control pulses were generated by this program to mark the onset of the pitch perturbation. Finally, the pitch-shifted voice signals were amplified by an ICON Neo Amp headphone amplifier and played back to participants through insert earphones (ER-1, Etymotic Research Inc.). The original and pitch-shifted voice signals as well as the TTL pulses were recorded at 10 kHz by a PowerLab A/D converter (model ML880, AD Instruments) using LabChart software (v.7.0, AD Instruments).

Simultaneously, the EEG signals were recorded using a 64-electrode Geodesic Sensor Net. They were amplified by a high input-impedance Net Amps 300 amplifier (Z_*in*_≈200 MΩ; Electrical Geodesics Inc.), and digitally sampled at 1 k Hz using NetStation software (v.4.5, Electrical Geodesics Inc.). For the synchronization of the voice and EEG signals, the TTL pulses were sent to the EEG recording system via an experimental DIN synch cable. The EEG signals across all channels were referenced to the vertex (Cz) during the online recording (Ferree et al., 2001). Since the amplifier accepts scalp-electrode impedances up to 60 kΩ (Ferree et al., 2001), the impedance levels of individual sensors were kept below 50 kΩ throughout the recording.

### Data Analyses

The magnitude and latency of vocal responses to pitch perturbations were measured using a custom-developed IGOR PRO software program (v.6.0 by WaveMetrics Inc.). First, the voice *f*_*o*_ contours in hertz were extracted from the voice signals using Praat software (Boersma, 2001) and converted to the cent scale using the following formula: cents = 100 × (12 × log_2_(*f*_*o*_/reference)) [reference = 195.997 Hz (G3)]. Next, the voice *f*_*o*_ contours were segmented into epochs ranging from 200 before to 700 ms after the onset of the pitch perturbation and visually inspected for artifact rejection. Individual trials contaminated by unexpected vocal interruptions or signal processing errors were regarded as bad trials and excluded from the following analyses. To examine the effects of LSVT LOUD on the compensatory mechanisms underlying auditory feedback control of vocal production in PD, only those trials that opposed the direction of the pitch perturbations were retained in the averaging analysis as other studies did (Guo et al., 2017; Scheerer and Jones, 2018; Patel et al., 2019). Overall, 56% and 58% of the individual trials were regarded as compensatory responses for individuals with PD and healthy controls, respectively. Finally, individual trials were averaged to generate an overall vocal response elicited by pitch perturbations. A baseline-correction procedure was applied to the averaged vocal responses by subtracting the mean *f*_*o*_ values in the baseline period (−200 ms to 0) from the *f*_*o*_ values after the perturbation onset. The magnitude in cents and latency in ms of a vocal response were separately measured as the greatest value and peak time when the voice *f*_*o*_ contour reached its maximum value.

The EEG signals were analyzed offline using NetStation software. First, they were band-pass filtered with cut-off frequencies of 1–20 Hz and segmented into epochs using a window of −200 to +500 ms relative to the onset of the pitch perturbation. Then, all segmented trials were submitted to an artifact detection procedure, during which those trials whose voltage values exceeded ±55 μv of the moving average over an 80-ms window were rejected from further analysis. An additional visual inspection was performed on a trial-by-trial level to ensure appropriate rejection of bad trials. Individual electrodes were rejected if they contained artifacts in more than 20% of the epochs, and files were marked bad if they contained more than 10 bad channels. Finally, after re-referenced to the average of the electrodes on each mastoid, artifact-free trials were averaged and baseline-corrected (−200 ms to 0) to generate an overall ERP response for each condition. Given that previous findings have shown robust and prominent cortical responses to pitch perturbations in the frontal and central regions (Scheerer et al., 2013; Huang et al., 2016), we chose 24 electrodes in three regions of interest (ROI) for statistical analysis: frontal area, including AF3, AFz, AF4, F5, F3, F1, Fz, F2, F4, and F6; fronto-central area, including FC5, FC3, FC1, FCz, FC2, FC4, and FC6; and central area, including C5, C3, C1, Cz, C2, C4, and C6. The amplitudes and latencies of N1 and P2 components, defined as the negative and positive peak values and times in the time windows of 80–180 and 160–280 ms relative to the perturbation onset, were extracted from the averaged ERP response for each ROI. Note that the EEG data of two individuals with PD (S5 and S7 in Table 1) were not recorded due to the technical problems. Thus, the EEG data from other 10 individuals with PD were compared with their one-to-one pair matched healthy controls in statistical analysis.

### Statistical Analyses

Prior to being submitted to SPSS (v.20.0) for statistical analyses, the original or log-transformed values of vocal and ERP responses to pitch perturbations were verified to be normally distributed using Kolmogorov–Smirnov tests. In order to determine the neural and behavioral differences in vocal pitch regulation between individuals with PD before LSVT LOUD and healthy controls, two samples *t*-tests were used to compare the magnitudes and latencies of vocal responses between two groups, while the amplitudes and latencies of the N1 and P2 responses were subjected to mixed-design analysis of variances (ANOVAs) with a between-subject factor of group and a within-subject factor of electrode site (frontal, fronto-central, and central). For the evaluation of neural and behavioral effects of LSVT LOUD on vocal pitch regulation in individuals with PD, the magnitudes and latencies of vocal responses were analyzed using paired samples *t*-tests, while the amplitudes and latencies of the N1 and P2 responses were subjected to repeated-measures ANOVAs (RM-ANOVAs) with two within-subject factors of vocal training (pre- vs. post-LSVT LOUD) and electrode site. Also, the values of vocal SPL for sustained phonation, passage reading, and monolog were analyzed using paired samples *t*-tests to determine the effects of LSVT LOUD on hypophonia. Bonferroni correction was used for multiple comparisons in *post hoc* analyses. The Greenhouse–Geisser was used to correct probability values for multiple degrees of freedom if the assumption of Mauchly’s test of sphericity for homogeneity of variance was violated. In order to quantify the proportion of variance, we calculated partial η^2^ (η${}_{\text{p}}^{2}$) as an index of effect size. The difference across the conditions was considered significant when *P*-values < 0.05 and η${}_{\text{p}}^{2}$ > 0.14 (Richardson, 2011).

## Results

**FIGURE 1:**
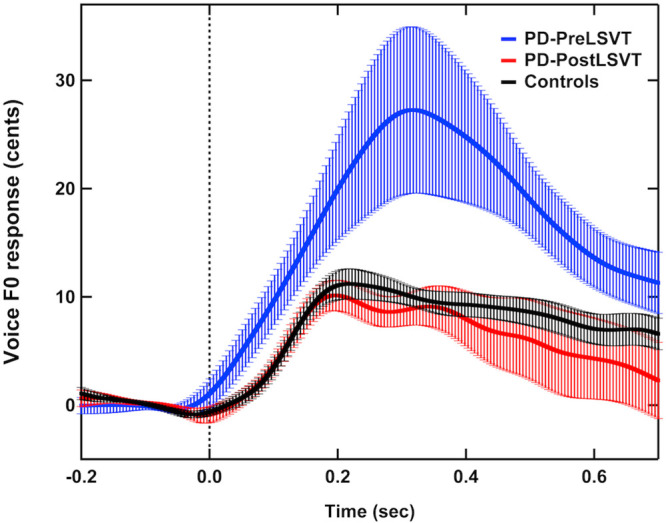
Grand-averaged voice *f*_*o*_ contours in response to pitch perturbations for individuals with PD before (red solid lines) and after (blue solid lines) LSVT LOUD and healthy controls (black solid lines). Highlighted areas indicate the standard errors of the mean vocal responses. Vertical dashed lines indicate the onset of the pitch perturbation.

**FIGURE 2:**
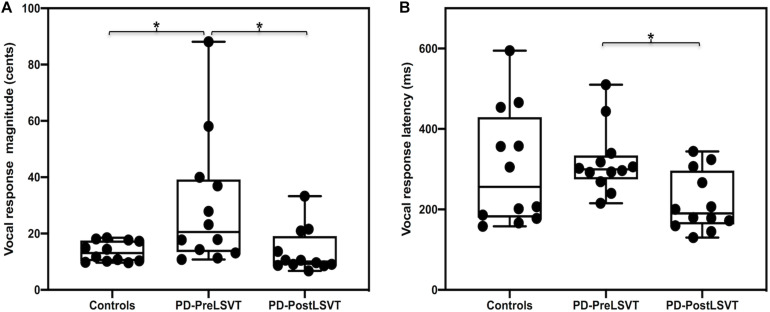
Box plots illustrating the medians and ranges of minimum and maximum magnitudes **(A)** and latencies **(B)** of vocal responses to pitch perturbations and individual data sets for individuals with PD before and after LSVT LOUD and healthy controls. The asterisks indicate that significant differences across the conditions.

Figure 1 shows the grand-averaged voice *f*_*o*_ contours in response to pitch perturbations for individuals with PD before and after LSVT LOUD and healthy controls. Figure 2 shows the boxplots with the medians and ranges from the minimum to maximum magnitudes and latencies of vocal responses as well as individual data sets across the conditions. As can be seen, individuals with PD before LSVT LOUD produced highly variable compensatory vocal responses than healthy controls, particularly for those from one participant (S10). His data, however, were kept for statistical analysis because they were still within the range of previously reported vocal responses produced by individuals with PD (Chen et al., 2013) and the results of significance tests remained unchanged regardless of whether his data were included.

**FIGURE 3:**
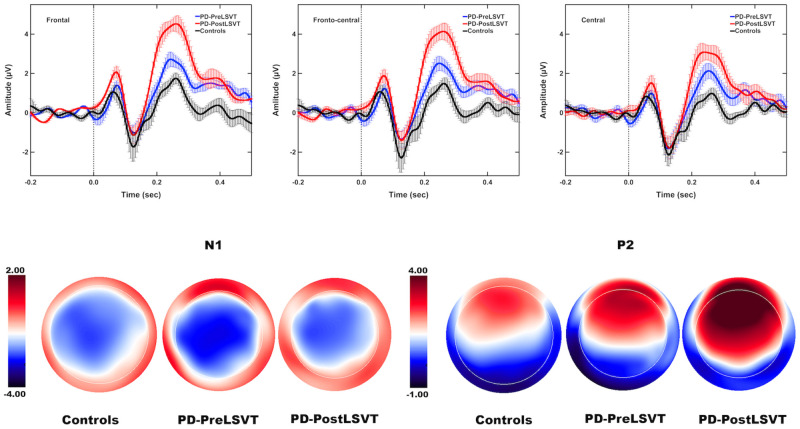
**Top:** Grand-averaged ERPs to pitch perturbations in the frontal (left), fronto-central (middle), and central (right) regions for individuals with PD before (red solid lines) and after (blue solid lines) LSVT LOUD and healthy controls (black solid lines). Highlighted areas indicate the standard errors of the mean ERPs. Vertical dashed lines indicate the onset of the pitch perturbation. **Bottom:** Topographical distribution maps of the grand-averaged N1 and P2 amplitudes in response to pitch perturbations for individuals with PD before (N1 latency: 129 ms; P2 latency: 248 ms) and after LSVT LOUD (N1 latency: 128 ms; P2 latency: 258 ms) and healthy controls (N1 latency: 127 ms; P2 latency: 257 ms).

**FIGURE 4:**
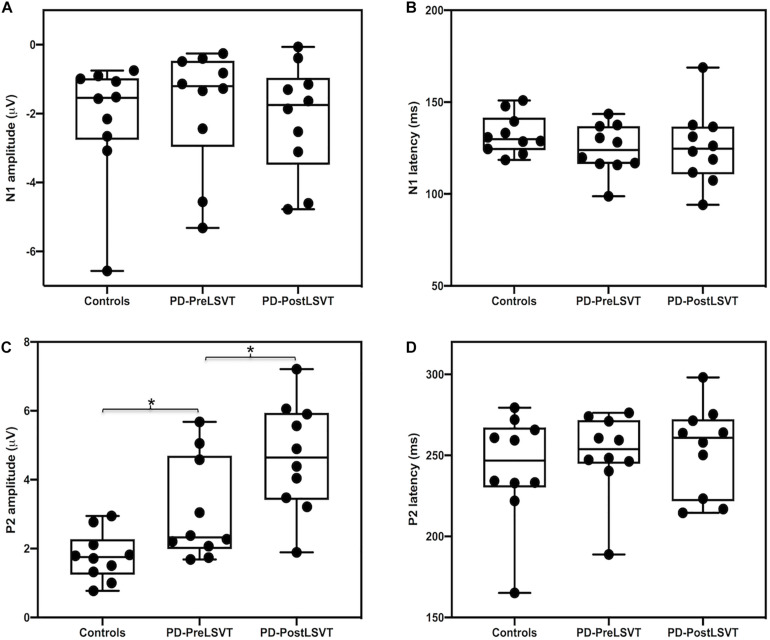
Box plots illustrating the medians and ranges from the minimum to maximum amplitudes and latencies of the N1 **(A,B)** and P2 **(C,D)** responses to pitch perturbations and individual data sets for individuals with PD before and after LSVT LOUD and healthy controls. The asterisks indicate significant differences across groups or conditions.

Figure 3 shows the grand-averaged ERP waveforms in response to pitch perturbations and topographical distributions of the grand-averaged N1 and P2 amplitudes across the conditions. Figure 4 shows the boxplots with the medians and ranges from the minimum to maximum amplitudes and latencies of the N1 and P2 responses as well as individual data sets across the conditions. As shown in Figures 3, 4, the effects of group and vocal training on the cortical processing of vocal pitch errors were primarily observed in the P2 component; individuals with PD produced larger P2 responses than healthy controls, and post-LSVT LOUD led to larger P2 amplitudes than pre-LSVT LOUD. In contrast, there were subtle changes in the N1 responses across the conditions.

### Pre-LSVT LOUD vs. Controls

When compared to one-to-one pair matched healthy controls, individuals with PD before LSVT LOUD produced significantly larger vocal compensations for pitch perturbations (*t* = 2.414, d.f. = 22, *p* = 0.025), indicating their impaired auditory-motor integration for vocal pitch regulation. The latencies of vocal responses, however, did not vary as a function of group (*t* = 0.342, d.f. = 22, *p* = 0.736).

A mixed-design ANOVA conducted on the N1 amplitudes revealed a significant main effect of electrode site [*F*(2,36) = 11.747, *p* = 0.001, η${}_{\text{p}}^{2}$ 0.395], indicating less negative N1 responses at the frontal electrodes relative to the central (*p* = 0.014) and fronto-central electrodes (*p* < 0.001). However, the main effect of group [*F*(1,18) = 0.269, *p* = 0.610] and its interaction with electrode site [*F*(2,36) = 1.081, *p* = 0.350] were not significant. In addition, the N1 latencies did not vary as a function of group [*F*(1,18) = 2.188, *p* = 0.156] and electrode site [*F*(2,36) = 1.130, *p* = 0.324]. The interaction between these two factors was not significant [*F*(2,36) = 0.567, *p* = 0.572].

By contrast, there was a significant main effect of group on the P2 amplitudes, showing larger P2 responses for individuals with PD than for one-to-one pair matched healthy controls [*F*(1,18) = 6.307, *p* = 0.022, η${}_{\text{p}}^{2}$ = 0.259] (see Figures 3, 4). A significant main effect of electrode site [*F*(2,36) = 14.376, *p* < 0.001, η${}_{\text{p}}^{2}$ = 0.444] was also found, resulting in smaller P2 amplitudes at the central electrodes relative to the frontal (*p* = 0.002) and fronto-central electrodes (*p* = 0.001). However, the interaction between group and electrode site was not significant [*F*(2,36) = 0.183, *p* = 0.834]. For the P2 latencies, there were no significant main effects of group [*F*(1,18) = 0.437, *p* = 0.517] and electrode site [*F*(2,36) = 1.520, *p* = 0.236]. Their interaction was not significant [*F*(2,36) = 0.268, *p* = 0.767].

### Pre- vs. Post-LSVT LOUD

Given that the present study included a partially overlapping participant cohort from our previous study (Li et al., 2020), the results of voice and speech measures were similar. Following LSVT LOUD, individuals with PD exhibited significantly improved vocal SPL during sustained phonation (66.32 ± 8.49 vs. 75.02 ± 3.96 dB; *t* = −4.450, d.f. = 11, *p* = 0.001), passage reading (64.44 ± 3.64 vs. 69.14 ± 5.42 dB; *t* = −3.950, d.f. = 11, *p* = 0.002), and monolog (61.04 ± 4.20 vs. 64.21 ± 3.96 dB; *t* = −3.188, d.f. = 11, *p* = 0.009), providing further evidence for efficacy of LSVT LOUD in the treatment of hypophonia.

The modulatory effects of LSVT LOUD on auditory-vocal integration in individuals with PD were assessed by comparing their vocal and ERP responses to pitch perturbation before and after LSVT LOUD. Post-LSVT LOUD led to significantly smaller vocal compensations than pre-LSVT LOUD (*t* = 3.648, d.f. = 11, *p* = 0.004) (see Figures 1, 2). There was also a significant main effect of vocal training (*t* = 3.043, d.f. = 11, *p* = 0.011) on the latencies of vocal responses, indicating that the time required to reach the peak vocal compensation was significantly shortened by LSVT LOUD (see Figures 1, 2).

At the cortical level, there was a significant main effect of electrode site on the N1 amplitudes [*F*(2,18) = 6.569, *p* = 0.007, η${}_{\text{p}}^{2}$ = 0.422], indicating more negative N1 responses at the fronto-central electrodes relative to the frontal electrodes (*p* = 0.014). However, the N1 amplitudes did not vary as a function of vocal training [*F*(1,9) = 0.300, *p* = 0.597]. The interaction between vocal training and electrode site was not significant [*F*(2,18) = 3.543, *p* = 0.073]. Regarding the N1 latencies, there were no significant main effects of vocal training [*F*(1,9) = 0.034, *p* = 0.857] and electrode site [*F*(2,18) = 2.192, *p* = 0.72]. Their interaction was not significant [*F*(2,18) = 1.210, *p* = 0.321].

By contrast, significantly larger P2 amplitudes were found when comparing post- vs. pre-LSVT LOUD [*F*(1,9) = 7.078, *p* = 0.026, η${}_{\text{p}}^{2}$ = 0.440] (see Figures 3, 4). Also, there was a significant main effect of electrode site [*F*(2,18) = 17.756, *p* = 0.001, η${}_{\text{p}}^{2}$ = 0.664], leading to smaller P2 amplitudes at the central electrodes relative to the frontal (*p* = 0.005) and fronto-central electrodes (*p* = 0.002). The interaction between vocal training and electrode site, however, was not significant [*F*(2,18) = 4.134, *p* = 0.065]. For the P2 latencies, there were no significant main effects of vocal training [*F*(1,9) = 0.044, *p* = 0.839] and electrode site [*F*(2,18) = 0.359, *p* = 0.703] as well as their interaction [*F*(2,18) = 0.931, *p* = 0.412].

**FIGURE 5:**
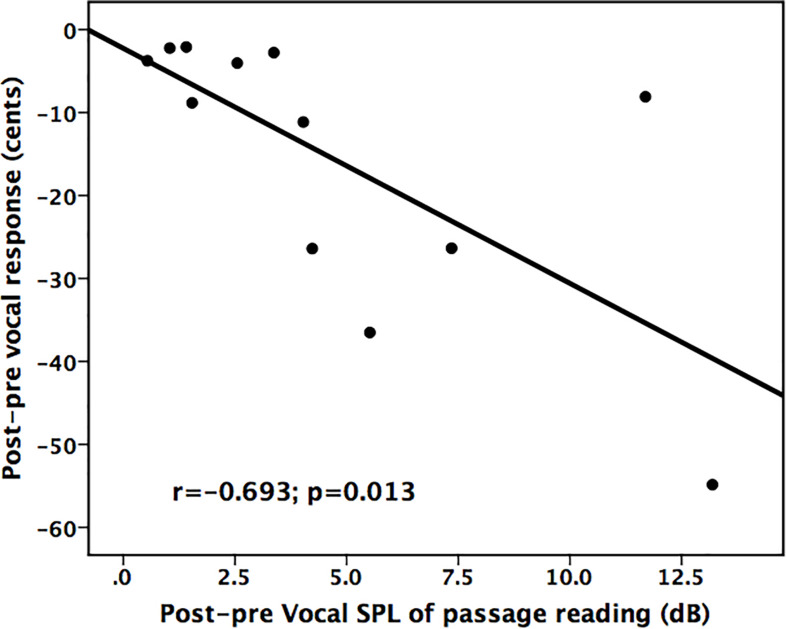
Scatter plots illustrating a significant correlation between improved vocal SPL during passage reading and reduced vocal compensations for pitch perturbations for individuals with PD following LSVT LOUD.

In order to examine whether treatment-induced improvement in vocal loudness contributed to changes in vocal pitch regulation, regression analyses were performed between changes in vocal SPL across the conditions and the post–pre differences in the vocal and ERP responses to pitch perturbations. As shown in Figure 5, there was a significant correlation between improved vocal SPL during passage reading and decreased vocal compensation magnitudes (*r* = −0.693, *p* = 0.013), indicating a transfer of beneficial effects of LSVT LOUD on vocal loudness to vocal pitch regulation in individuals with PD.

## Discussion

The present study investigated whether impaired auditory-motor control of vocal pitch production in PD can benefit from intensive voice treatment with LSVT LOUD. The results replicated earlier reports that individuals with PD exhibited enhanced vocal and P2 responses to pitch perturbations relative to healthy controls and improved vocal SPL during sustained phonation, passage reading, and monolog following LSVT LOUD. Most importantly, compensatory vocal responses became significantly smaller and faster, while P2 responses became significantly larger following LSVT LOUD. Moreover, the extent of improved vocal SPL during passage reading was significantly correlated with the degree of reduced vocal compensations for pitch perturbations. These findings provide the preliminary evidence for beneficial effects of LSVT LOUD on impaired auditory-motor integration for vocal pitch regulation associated with PD at the behavioral and neural levels.

### The Impact of PD on Auditory-Vocal Integration

Consistent with previous behavioral studies (Liu et al., 2012; Chen et al., 2013; Huang et al., 2016; Mollaei et al., 2016), the present study showed larger vocal compensations for pitch perturbations in individuals with PD relative to healthy controls. Also, the observation of enhanced cortical P2 responses to pitch perturbations associated with PD was consistent with previous findings reported by Huang et al. (2016), in which this pattern of cortical activity was related to increased activity in the left superior temporal gyrus (STG), inferior parietal lobule (IPL), inferior frontal gyrus (IFG), and PMC. Consistently, several neuroimaging studies have identified greater activation of these cortical regions during speech production in individuals with PD when compared to healthy controls (Liotti et al., 2003; Pinto et al., 2004; Arnold et al., 2014). It is thus suggested that impairments in auditory-vocal integration in individuals with PD may be attributed to hyperactivity in their cortical speech motor networks (Huang et al., 2016). This idea is supported by one recent study showing that improved speech articulation in individuals with PD after brain activity in the right STG was depressed by low-frequency repetitive transcranial magnetic stimulation (rTMS) (Brabenec et al., 2019).

### Neurobehavioral Effects of LSVT LOUD

While replicating earlier reports of improved vocal SPL following LSVT LOUD (Ramig et al., 2001b; Fox et al., 2012; Sauvageau et al., 2015; Nakayama et al., 2020), the present study, for the first time, revealed the behavioral effects of LSVT LOUD on self-monitoring of vocal pitch production in individuals with PD as reflected by their smaller and faster vocal compensations for pitch perturbations. Of particular interest, the degree of reduced vocal response magnitudes was predictive of the amount of improvement in vocal SPL during passage reading, suggesting that LSVT LOUD on hypophonia can produce positive transfer effects to facilitating auditory-motor integration for vocal pitch regulation. Also, individuals with PD exhibited significantly larger P2 responses to voice pitch perturbations when comparing post- and pre-LSVT LOUD. This enhancement of cortical activity is in line with one neuroimaging study by Narayana et al. (2010), in which individuals with PD exhibited increased activity in the right primary motor cortex (M1), STG, IPL, and DLPFC that was significantly correlated with improved vocal SPL following LSVT LOUD. Similarly, the present findings are in line with another study showing reduced vocal compensations for pitch perturbations that were accompanied by with enhanced P2 responses in individuals with PD following external auditory cueing (Huang et al., 2019), a behavioral approach that is effective in increasing their vocal pitch and loudness levels and improving speech intelligibility and articulatory movement (Dromey and Ramig, 1998; Goberman and Elmer, 2005). Together, these neurobehavioral changes in individuals with PD following LSVT LOUD may represent an improvement of their ability to appropriately detect and/or correct auditory feedback errors for controlling vocal pitch production that may be related to a functional reorganization of speech motor networks.

It is noteworthy that LSVT LOUD did not lead to systematic changes of N1 responses to pitch perturbations. This is in contrast with other studies that have shown decreased N1 responses to pitch perturbations in healthy participants following speech perceptual learning and auditory working memory training (Chen et al., 2015; Li et al., 2015) and individuals with PD following external auditory cueing (Huang et al., 2019), reflecting increased efficiency in the neural encoding of pitch information in auditory feedback (Zatorre et al., 2012). Although we cannot provide specific explanations for the absence of N1 modulation following LSVT LOUD, it may be related to the differences in the training protocol. In previous studies (Chen et al., 2015; Li et al., 2015; Huang et al., 2019), the participants were required to learn to perceive different lexical tones, remember digits with varying signal-noise-ration (SNR) levels, or vocalize to match specific pitch target, which demands extensive involvement of auditory-related regions that contributed to the generation of N1 responses. In contrast, LSVT LOUD is specifically designed to improve vocal SPL for speech tasks that has been found to accompanied with changes in cortical activity of motor and premotor regions as well as the DLPFC (Liotti et al., 2003; Narayana et al., 2010), which may not lead to the modulatory effects on the N1 responses.

### Potential Mechanisms of Efficacy of LSVT LOUD

The results from the present and previous studies (Liu et al., 2012; Chen et al., 2013; Huang et al., 2016; Mollaei et al., 2016) have shown enhanced vocal and/or cortical P2 responses to pitch perturbations in individuals with PD relative to healthy controls. These abnormalities have been thought to be related to their deficits in laryngeal control systems and dysfunctions in the speech motor networks (Hammer and Barlow, 2010; Chen et al., 2013; Huang et al., 2016). Therefore, the observed beneficial effects of LSVT LOUD on auditory-vocal integration in PD can be discussed from two perspectives: (1) improved laryngeal motor functions and (2) a top-down modulation of vocal motor behaviors.

Evidence from peripheral studies has shown the importance of groups of laryngeal muscles and status of the vocal folds for precise voice *f*_*o*_ control. For example, anesthetization of the vocal folds led to increased vocal compensations for pitch perturbations (Larson et al., 2008), and the cricothyroid and thyroarytenoid muscles changed their activity in the same direction as that of vocal responses to pitch perturbations (Liu et al., 2011a). Deficits of laryngeal control systems associated with PD have been well documented, including decreased closure of the vocal folds, increased laryngeal resistance, and reduced activity in laryngeal muscle (Ramig and Dromey, 1996; Baker et al., 1998; Luschei et al., 1999; Ramig et al., 2004; Hammer and Barlow, 2010). Following LSVT LOUD, however, individuals with PD exhibited improvements in vocal fold adduction, vocal fold vibratory movements, and increased activity in thyroarytenoid muscle (Fox et al., 2002; Mahler et al., 2015). While contributing to increased vocal loudness, *f*_*o*_ range, and articulatory precision, these physiologic changes may also facilitate auditory-motor integration for compensatory adjustments of vocal motor behaviors with precision.

In addition to reduced vocal compensations for pitch perturbations, LSVT LOUD also led to increased cortical P2 responses. This brain–behavior relationship may reflect a top-down modulatory effect of LSVT LOUD on vocal motor control. Previous research has shown an association between reduced vocal compensations for pitch perturbations and increased P2 amplitudes in healthy populations following working memory training (Guo et al., 2017) and in individuals with PD following external auditory cueing (Huang et al., 2019). Moreover, one recent TMS study reported that disrupting activity in the left DLPFC by continuous theta-burst stimulation (c-TBS) led to enhanced vocal compensations for pitch perturbations that were accompanied by reduced P2 responses (Liu et al., 2020). Consistently, reduced high-gamma activity in the DLPFC was predictive of abnormally enhanced vocal compensations for pitch perturbations in patients with AD (Ranasinghe et al., 2019). In addition, recent source localization work has shown the contribution of the prefrontal cortex to the generation of P2 responses to pitch perturbations (Huang et al., 2016; Guo et al., 2017). It is thus suggested that a top-down inhibitory mechanism mediated by the prefrontal cortex may underlie auditory-motor control of vocal production, preventing vocal motor behaviors from being excessively influenced by auditory feedback (Liu et al., 2020). In light of this idea, individuals with PD may be impaired in this top-down mechanism such that auditory feedback errors cannot be correctly perceived and appropriately corrected, resulting in their abnormally enhanced vocal compensations. Therefore, our observation of enhanced P2 responses may reflect a functional reorganization of speech vocal networks by LSVT LOUD, restoring a normal activation of this top-down mechanism to exert inhibitory control over auditory-vocal integration that led to reduced vocal compensations. This hypothesis is in line with two other studies that reported a significant correlation between increased activity in the DLPFC and improved vocal SPL observed and decreased activation in the motor/premotor regions in individuals with PD following LSVT LOUD (Liotti et al., 2003; Narayana et al., 2010).

Given previously reported hyperactivity in the speech motor networks in individuals with PD during speech production (Liotti et al., 2003; Pinto et al., 2004; Rektorova et al., 2007; Huang et al., 2016), one might predict a decrease of cortical brain activity that is accompanied by improved auditory-vocal integration. This is in contrast with increased cortical activity following LSVT LOUD observed in the present and previous studies (Narayana et al., 2010; Baumann et al., 2018). However, there is evidence showing that improved hypokinetic dysarthria by LSVT LOUD is accompanied by increased activation in the right DLPFC, anterior insula, and basal ganglia and decreased activation in the motor/premotor regions (Liotti et al., 2003). In addition, individuals with PD exhibited an overactivation of the DLPFC and insula and deactivation of the motor regions during speech production that were reversed following subthalamic nucleus stimulation (Pinto et al., 2004). These findings indicate that the neural bases of speech disorders associated with PD are more complex that are often assumed. Although very speculative, enhanced P2 responses observed in the present study might represent activity from multiple neural sources (Huang et al., 2016) for a normalization of the top-down mechanism following LSVT LOUD, recruiting more prefrontal sources to inhibit motor-premotor activity that reduces overcompensation in response to vocal pitch errors in individuals with PD. This hypothesis needs to be tested in further work using neuroimaging techniques.

Note that a new approach termed LSVT BIG targeting increased movement amplitude has been developed for the treatment of the hypokinesia and bradykinesia in PD (Fox et al., 2012). There is evidence showing improvement in motor functions for individuals with PD following LSVT BIG (Ebersbach et al., 2010; McDonnell et al., 2018; Peterka et al., 2020), which may also be related to training-induced neuroplasticity. Although both speech and limb movement are complex motor skills, considerable evidence has shown distinct neural mechanisms underlying disorders of these two functions associated with PD. For example, increased activation in the fronto-tempo-parietal network has been identified in individuals with PD during speech production (Liotti et al., 2003; Narayana et al., 2010; Huang et al., 2016), whereas they exhibited hypoactivity in the DLPFC and SMA but hyperactivity in the PMC during movement (Playford et al., 1992; Haslinger et al., 2001; Yu et al., 2007). Therefore, whether top-down modulations can augment movement control as they do for speech production remains unclear, and more studies are needed to elucidate the neural mechanisms underlying the role of exercise innervation in treating movement disorders in PD.

### Limitations

Several limitations of the present study should be addressed. First of all, the sample size is small in the present study. This is due to the fact that speech disorders receive much less attention and are often ignored as compared to motor symptoms in Chinese individuals with PD, leading to difficulty in recruiting enough participants for LSVT LOUD. Second, the lack of an untreated PD group in the present study led to a possibility that the present findings may be the results of placebo induced improvement. On the other hand, the present study did not examine the long-term effects of LSVT LOUD in Chinese individuals with PD because of their difficulty in visiting a hospital due to geographical issues. Future studies, therefore, should include larger sample size to confirm the robustness of beneficial effects of LSVT LOUD and an untreated PD group to single out confounding factors of treatment specificity and test–retest reliability. Notwithstanding these limitations, the present study provides preliminary evidence for linking LSVT LOUD to impaired auditory-motor integration for vocal pitch regulation in individuals with PD.

## Conclusion

The present study revealed beneficial effects of LSVT LOUD on auditory-vocal integration in individuals with PD, as reflected by reduced vocal compensations that were predictive of improved vocal SLP during passage reading and increased cortical P2 amplitudes in response to pitch perturbations in auditory feedback. These neurobehavioral changes may be related to improved laryngeal control functions and a top-down modulation of vocal motor behaviors following LSVT LOUD. These preliminary findings provide evidence suggesting that LSVT LOUD on vocal loudness can produce positive transfer effects to facilitating auditory-motor integration for vocal pitch regulation in individuals with PD.

## Data Availability Statement

The original contributions presented in the study are included in the article/supplementary material. Further inquiries can be directed to the corresponding author/s.

## Ethics Statement

The studies involving human participants were reviewed and approved by the Institutional Review Board of The First Affiliated Hospital at Sun Yat-sen University. The patients/participants provided their written informed consent to participate in this study.

## Author Contributions

YL, MT, XC, and HL designed the experiments. YL, MT, HF, JL, and XC performed the experiments and analyzed the data. YL, EW, LC, XC, and HL interpreted the results and wrote the manuscript. All authors read and approved the final manuscript.

## Conflict of Interest

The authors declare that the research was conducted in the absence of any commercial or financial relationships that could be construed as a potential conflict of interest.
